# Priorities for methodological research on patient and public involvement in clinical trials: A modified Delphi process

**DOI:** 10.1111/hex.12583

**Published:** 2017-06-15

**Authors:** Anna Kearney, Paula Williamson, Bridget Young, Heather Bagley, Carrol Gamble, Simon Denegri, Delia Muir, Natalie A. Simon, Stephen Thomas, Jim T. Elliot, Helen Bulbeck, Joanna C. Crocker, Claire Planner, Claire Vale, Mike Clarke, Tim Sprosen, Kerry Woolfall

**Affiliations:** ^1^ Clinical Trials Research Centre (CTRC) North West Hub for Trials Methodology University of Liverpool Liverpool UK; ^2^ Department of Psychological Sciences North West Hub for Trials Methodology University of Liverpool Liverpool UK; ^3^ School of Life and Medical Sciences University College London London UK; ^4^ Leeds Institute of Clinical Trials Research (LICTR) University of Leeds Leeds UK; ^5^ Public Involvement and Engagement Health Care Research Wales Cardiff UK; ^6^ Nuffield Department of Primary Care Health Sciences Health Experiences Institute University of Oxford Oxford UK; ^7^ Centre for Primary Care University of Manchester Manchester UK; ^8^ MRC Clinical Trials Unit University College London London UK; ^9^ Centre for Public Health Queen's University of Belfast Belfast UK; ^10^ Nuffield Department of Population Health Oxford University Oxford UK

**Keywords:** clinical trials, Delphi, patient and public involvement, research priorities

## Abstract

**Background:**

Despite increasing international interest, there is a lack of evidence about the most efficient, effective and acceptable ways to implement patient and public involvement (PPI) in clinical trials.

**Objective:**

To identify the priorities of UK PPI stakeholders for methodological research to help resolve uncertainties about PPI in clinical trials.

**Design:**

A modified Delphi process including a two round online survey and a stakeholder consensus meeting.

**Participants:**

In total, 237 people registered of whom 219 (92%) completed the first round. One hundred and eighty‐seven of 219 (85%) completed the second; 25 stakeholders attended the consensus meeting.

**Results:**

Round 1 of the survey comprised 36 topics; 42 topics were considered in round 2 and at the consensus meeting. Approximately 96% of meeting participants rated the top three topics as equally important. These were as follows: developing strong and productive working relationships between researchers and PPI contributors; exploring PPI practices in selecting trial outcomes of importance to patients; and a systematic review of PPI activity to improve the accessibility and usefulness of trial information (eg participant information sheets) for participants.

**Conclusions:**

The prioritized methodological research topics indicate important areas of uncertainty about PPI in trials. Addressing these uncertainties will be critical to enhancing PPI. Our findings should be used in the planning and funding of PPI in clinical trials to help focus research efforts and minimize waste.

## 1 INTRODUCTION

Growing awareness of the importance of patient centeredness in research^1^, ^2^ has influenced the establishment of Patient‐Centred Outcomes Research Institute in the United States, the National Institute for Health Research (NIHR) INVOLVE organization in the United Kingdom (UK) and similar bodies elsewhere. These organizations have been at the vanguard of international efforts to involve patients as research partners, alongside researchers, to set research agendas, design studies and decide what outcomes should be measured.^3^, ^4^ The emphasis on patient centeredness in research stems from a belief that involving patients in decisions about how studies are designed and conducted improves research, making it more relevant to end users^3^, ^5^, ^6^, ^7^ and reducing waste.^8^, ^9^ Patient involvement is also believed important for moral reasons, based on the principle that the people whose lives are most affected by research should have a say. In the UK, patient involvement is known as patient and public involvement (PPI).^5^, ^10^ In clinical trials, PPI tends to involve a small number of patients or members of the public (known as PPI contributors).^11^ Some PPI contributors will have direct personal experience of the condition being investigated, whilst others bring general experience of being a patient or service user. A key consideration is that PPI contributors are in a position to offer a distinctive perspective to researchers or clinicians. Many UK funders require researchers seeking funding to provide evidence of how PPI will inform their studies.^12^, ^13^, ^14^

Despite the emphasis on PPI in the UK and internationally, there are uncertainties about how best to implement it,^15^ about the purpose of PPI and whether it actually does improve research.^10^, ^12^, ^15^, ^16^ Concerns have been raised about tokenism and resourcing in PPI, about the difficulty of ensuring diversity and avoiding professionalization among PPI contributors,^10^, ^17^, ^18^ complexities with researchers and patients sharing power,^19^ and inadequacies in training and support for both PPI contributors and researchers.^20^ Problems with the conceptualization and meaningful assessment and measurement of PPI have also been identified.^21^

Each of these concerns points to different priorities for methodological research on PPI. Reviews of PPI in research and other contexts identify many topics for future research.^4^, ^21^, ^22^, ^23^, ^24^ Although not all reviews focus specifically on clinical trials, trials are regarded as particularly likely to benefit from PPI^20^, ^25^ by helping to address the many methodological issues that arise within trials.^5^ Most reviews of PPI echo similar concerns to those identified in the above paragraph, pointing to the need for: agreed tools for measuring PPI and its impact across the different phases of research,^15^, ^24^, ^26^, ^27^ for investigations of how best to support PPI^6^, ^23^, ^28^ and for optimal models of implementing PPI.^29^, ^30^ However, many of these topics have been identified by PPI researchers and it is unclear whether these priorities are shared by the wider community of trialists and PPI stakeholders. Given the diversity of stakeholders involved in PPI, there is considerable potential for divergence in the prioritization of topics to investigate, and therefore for dilution of research efforts in investigating how to improve PPI in research.

In the **METHOD**s for Patient and Public **I**nvolvement In **C**linical Tri**AL**s (METHODICAL study), we conducted a modified Delphi process to identify the priorities of a broad range of PPI stakeholders for methodological research to resolve uncertainties about PPI in clinical trials, as well as to help improve to the design of future PPI research and avoid unnecessary duplication of research effort.

## 2 MATERIALS AND METHODS

Delphis are used in health and social science research as a means of involving participants with relevant experience, via a multistaged study, to achieve consensus on a given topic.^16^, ^31^, ^32^ This involves conducting sequential anonymous surveys to collect, collate and present results back to the group. To help achieve consensus, participants can view and revise their own responses in the light of group responses.^32^ The process can be modified to include opportunities for feedback or a consensus meeting so that participants can discuss their views.^33^, ^34^ We designed a modified Delphi, comprising a literature review to identify topics for research on PPI, followed by a two round online survey and stakeholder consensus meeting.

We established a study team of 17 PPI stakeholders from across the UK to oversee the METHODICAL project, including: four PPI Coordinators, eight PPI researchers, one PPI planner, two PPI contributors, one non‐lay reviewer and one lay reviewer. Seven members of the team had secondary PPI‐related roles.

### 2.1 Patient involvement

Our study team included three patient partners who were involved in all aspects of study design and conduct, including development of protocol, pilot topics and accompanying text, survey recruitment, interpretation of study findings and review of this manuscript. Approximately half of the consensus meeting places were allocated to patients. We will send participants a summary of the study findings. The summary will also be placed on the study website and promoted through social media platforms used by patients.

### 2.2 Recruitment

To help maximize the utility of our findings, we aimed to include all key paid and unpaid roles of people who co‐ordinate, support and contribute to PPI in trials. Individuals were eligible to participate in the Delphi process if they had at least 12 months’ experience within a PPI role in clinical trials. Study team members did not participate in the survey. As definitions of roles in PPI vary, the study team identified seven stakeholder groups to inform recruitment, consulting with our PPI partners to select terminology to define each group (Table 1). We provided this list of stakeholder groups and accompanying definitions in recruitment materials. A free text field was included at registration so participants could elaborate on their role/s and self‐identify their role if they felt this was not included in the list. The study team agreed that for the feedback of results in round 2 to be meaningful at the level of stakeholder group, approximately 10 participants per group would be required.

**Table 1:** Stakeholder groups for the Delphi process

Stakeholder Group	Definition and examples
PPI Contributors	Patient representatives, research partners in clinical trials
Lay Reviewers	Members of the public sitting on clinical trial funding boards or Research Ethics Committees (RECs)
PPI Coordinators	Roles within a clinical trial unit (CTU) or research network to coordinate PPI activity and PPI contributors and research partners in trials
PPI Advisors	Roles offering advice on how to design and deliver PPI activity within trials. This predominantly includes member of the National Institute for Health Research (NIHR) Research Design Service (RDS)
PPI Planners	Chief Investigators, trial managers and other researchers/staff who plan or oversee PPI in individual trials
PPI Researchers	People who conduct research into PPI in clinical trials and authors of PPI guidance documents
Non‐lay Reviewers	Professional members of clinical trial funding boards or Research Ethics Committees (RECs)

We used snowball sampling to identify stakeholders^35^ using personal contacts and Internet searches to develop a database of individuals, organizations and networks under each of the seven stakeholder groups. The METHODICAL researcher (AK) sent emails to the identified organizations, networks and individuals (Table S1) with study information. The email included a request to invite potential survey participants by distributing the study invitation to their members or contacts. AK also placed an advert and link to the survey on the “People in Research Forum”(www.peopleinresearch.org).

### 2.3 Development and pilot of topics

**Figure 1:**
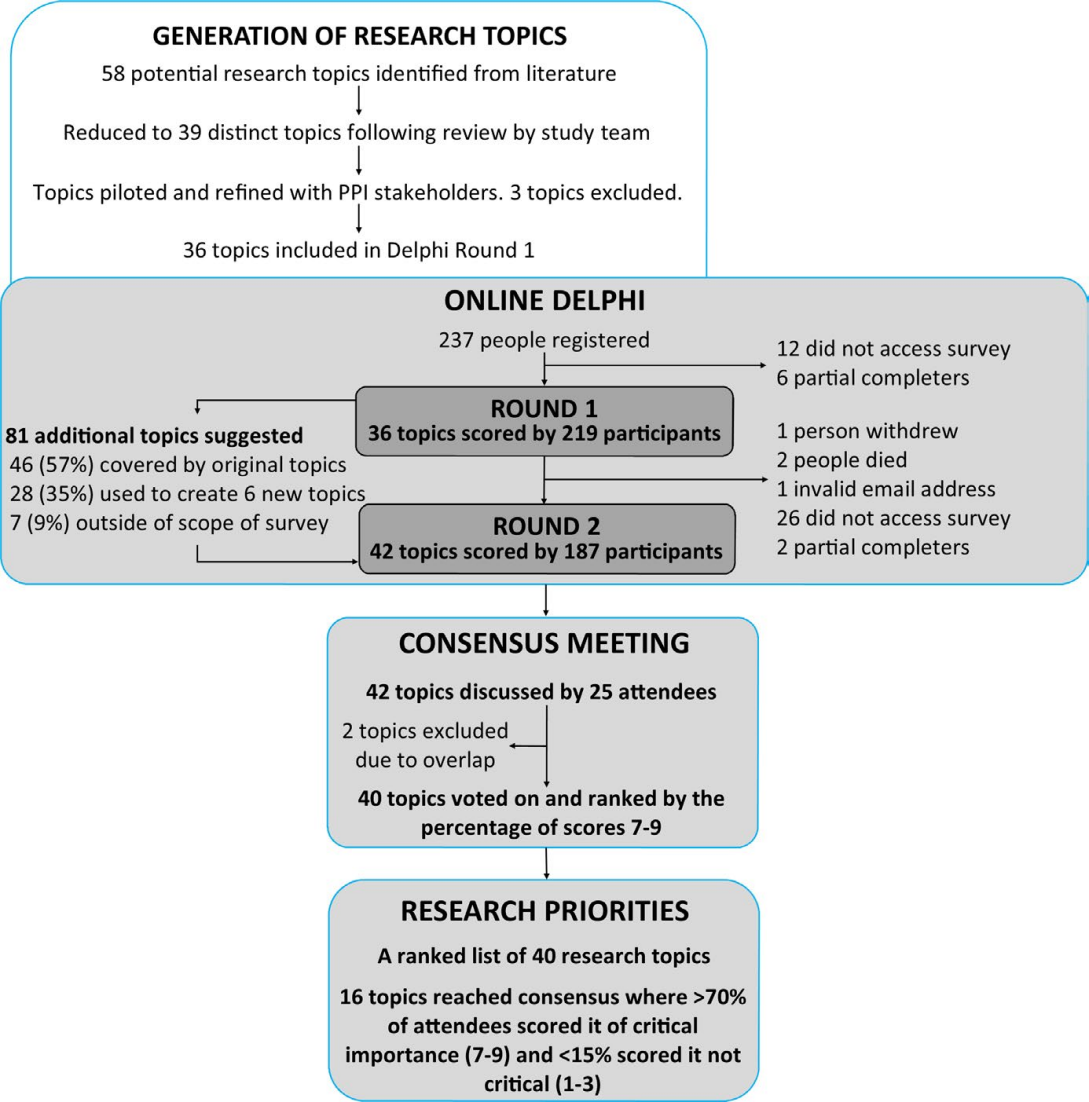
Overview of the Delphi process

We used online search engines (eg Google Scholar and OVID (MEDLINE), organizational databases (eg INVOLVE library) and hand searches of citations within key articles to identify literature that systematically evaluated the scope and impact of PPI within health research^15^, ^20^, ^24^ to develop a list of potential methodological research topics for round 1 of the Delphi. This was supplemented by reviewing recent publications assessing PPI specifically within clinical trials.^22^, ^23^, ^27^, ^36^ For each topic, we developed accompanying descriptive text to help explain these. The study team, including PPI partners reviewed the list of topics and accompanying descriptions to ensure they were distinct and clearly communicated challenges associated with PPI in clinical trials. Methodological research in this context was described to participants in study information materials as: “methods, practices and procedures of PPI in clinical trials.” We piloted the list of topics with a small group of lay (n=2) and non‐lay (n=3) PPI stakeholders to check clarity and understanding and then refined the list of topics and descriptive text (Figure 1).

### 2.4 Online survey

The online Delphi was conducted between November 2015 and March 2016. Round 1 was open for approximately 5 weeks and round 2 for 4.5 weeks.

In round 1, stakeholders registered for the study by indicating their name, email address, which of the seven stakeholder groups they had the most experience in, years of PPI experience, consent to participate, interest in attending the consensus meeting and interest in receiving a copy of the published findings. We assigned each registered user a unique identifier to ensure anonymity and enable linking of scores between rounds. Participants then scored the importance of each of research topic using a scale of 1‐9, with scores 1‐3 being not critical or low importance, 4‐6 important but not critical and 7‐9 of critical importance.^37^ Selecting a score of 10 indicated an abstention from scoring an individual topic. Participants were also invited to suggest additional topics to be added to round 2. Participants who registered but did not start the survey, or partially completed round 1 questions, were excluded from the analysis and not invited for round 2. The study team reviewed additional topics suggested by participants in round 1 for inclusion in round 2.

In round 2, we showed participants bar charts summarizing the distribution of the percentage of scores 1‐9 for each topic from each stakeholder group. We then invited participants to revise or keep their own score from the previous round. The email invitation for round 2 indicated that responses received within 10 or 17 days would be entered into prize draws for a £50 voucher or a £30 voucher, respectively. AK sent email reminders periodically to non‐responders.

### 2.5 Consensus meeting

We allocated thirty places to equal numbers of lay and non‐lay stakeholders with broad representation across the seven stakeholder groups. The METHODICAL study team were invited to attend and participate in the consensus meeting. Three study team members helped to facilitate the meeting and did not take part. Ten other study team members registered to attend as participating stakeholders and were allocated either lay or non‐lay places based on their primary PPI roles. We invited survey participants at random within their stakeholder group. Only survey participants who completed both rounds of the survey and who registered their interest in attending the consensus meeting were eligible to attend.

AK emailed each registered attendee a copy of the agenda and their scores from round 2 one week before the meeting. PW, a member of the METHODICAL team, facilitated the meeting due to her previous experience in this role. PW had no vested interests in the ranking of research priorities, although like all survey participants, she is involved in the design and delivery of clinical trials. Team members KW and AK began the meeting with a short‐study overview. KW presented the results from round 2 sequentially and in the same order as presented in the online survey. Each topic and accompanying description were presented together with bar charts showing how each stakeholder group had scored each topic. We provided attendees with paper copies of their individual scores and the level of consensus achieved within stakeholder groups during round 2 (Table S4). PW began by asking attendees if any clarification of the topic was required. Comments and discussion were then encouraged before PW asked attendees to consider whether or not the topic should be prioritized for future research. Where more than 70% of round 2 participants in any one stakeholder group had indicated a topic was of high importance (scored it 7‐9), we invited attendees to raise opposing arguments. A similar approach was followed for those topics where less than 50% of round 2 participants in any one group had indicated a topic to be of less importance (scored it 1‐3), with views requested if a participant felt strongly that a topic should be considered important. PW encouraged a fuller discussion where the online survey results indicated mixed views on a topic. Following discussion of each topic an anonymous vote was undertaken using a hand held voting device (Turning Point software, version 5, Turning Technologies, youngstown, Ohio, USA). Meeting attendees could abstain from voting for an individual topic by selecting a score of 10. This process was repeated until all topics were discussed and voted on.

AK circulated a written report to meeting attendees seven weeks after the meeting, which included notes from meeting discussions and any changes made to the topic description text.

### 2.6 Statistical analysis

We pre‐defined consensus as 70% or more participants scoring from 7 to 9 and less than 15% participants scoring from 1 to 3 on a particular topic.^38^, ^39^ All statistical analysis was performed in R version 3.2 (R Foundation for Statistical Computing , Vienna, Austria.www.R-project.org/).We ranked final research topics from the METHODICAL consensus meeting according to the percentage of participants scoring a research topic as critically important (scores 7‐9) and then by ascending order of the percentage of scores 1‐3.

## 3 RESULTS

### 3.1 Online survey

Of the 237 people who registered for the survey, 219 (92%) completed round 1. Twelve individuals registered but did not start the survey and six provided partial responses (Figure 1). All 18 individuals were excluded from the analysis. Of the 219 who completed round 1, 187 (85%) completed round 2 and were included in the analysis. Of the remaining 32, one withdrew from round 2 of the survey, two died, two partially completed round 2, and 27 did not complete any part. Completion rates by stakeholder group for round 1 and 2 are shown in Table 2. Round 1 of the survey comprised 36 methodological research topics (Table S2). The study team reviewed 81 additional research topics suggested by survey participants. Of these, we agreed that 46 suggestions were within the scope of existing topics, although we added additional examples to seven existing topics or descriptors to improve their clarity. Twenty‐eight suggestions contributed to the development of six new topics which were added to round 2. The remaining seven suggestions related to trial participants not PPI and were therefore considered to be out of scope. However, these led to the inclusion of a new topic aimed at exploring the definition of PPI and people's understanding of it. Round 2 of the survey comprised of 42 methodological research topics, including the six new topics created from participant suggestions.

**Table 2:** Stakeholder representation within the survey and at the meeting

Stakeholder group	No. Registered	No. who completed round 1 (% of registered^a^)	No. who completed round 2 (% of round 1)	No. who accepted the meeting invitation (% of round 2 completers)	No. of meeting attendees (% of those invited)
Lay reviewers	51	48 (94)	39 (81)	7 (18)	6 (86)
PPI contributors	37	36 (97)	27 (75)	8 (30)	6 (75)
Total Lay	88	84 (95)	66 (79)	15 (23)	12 (80)
Non‐lay reviewers	40	38 (95)	33 (87)	3 (9)	3 (100)
PPI Planners	53	47 (89)	39 (83)	4 (10)	4 (100)
PPI advisors	13	12 (92)	12 (100)	1 (8)	0^b^ (0)
PPI coordinators	26	25 (96)	25 (100)	4 (16)	4 (100)
PPI researchers	17	13 (76)	12 (92)	3 (25)	2 (67)
Total Non‐lay	149	135 (91)	121 (90)	15 (12)	13 (87)
Total	237	219 (92)	187 (85)	30 (16)	25 (83)

a For example, the percentage of Lay reviewers who registered and completed round 1 (94%) is the number who completed (n=48) divided by the number registered (n=51).

b At least two people with secondary roles of PPI advisor were present at the consensus meeting.

At the end of round 2, we reviewed results against the definition of consensus agreed at the beginning of the study. At the end of round 2, there was no consensus across all stakeholder groups as to which research topics were of critical importance. Only three topics achieved consensus across six of the seven groups (Table S4).

### 3.2 Consensus meeting

Of the 30 people registered, 25 attended and were eligible to vote (Table 2). Seventeen were survey participants and eight were members of the METHODICAL study team. Twelve (48%) attendees were lay, and 13 (52%) were non‐lay. Although no attendees identified PPI advisor as the stakeholder group that they most identified with, at least two had PPI advisor roles; all stakeholder groups were, therefore, represented at the meeting.

All 42 topics were discussed, and voting was undertaken on all except two, topics 38 and 39. Following discussion attendees concluded that topic 38 (methods to measure PPI impact) should be subsumed within topic 37 (core outcomes to evaluate PPI), while topic 39 (characteristics of PPI which lead to a successful trial) was considered to be too broad. We made changes to three topic titles and nine descriptive texts after group discussion in order to clarify the topic before voting (Table S2).

Table S3 provides the final ranked list of all research topics. Sixteen topics achieved consensus with greater than 70% of participants scoring them 7‐9 and less than 15% scoring them 1‐3. As shown in Table 3, the top 10 prioritized research topics were varied, covering PPI processes, resources, practices and relationships between stakeholder groups. Three topics shared joint “first place” with 96% of meeting attendees rating each as critically important: developing strong and productive working relationship between researchers and PPI contributors; PPI practices in selecting trial outcomes of importance to patients; and a systematic review of PPI activity in improving the accessibility and usefulness of trial information (eg leaflets and information sheets) for clinical trial participants.

**Table 3:** Top 10 Methodological priorities for PPI in clinical trials

Ranking	Topic No.	Topic Title	Help text	% of meeting scores	
				7‐9 (%)	1‐3 (%)
1	TOPIC 20	Developing strong and productive working relationships between researchers and PPI contributors	Research on what defines and enables a good working relationship between researchers on a trial team, trial committee (eg trial steering committee or ethics committee) or funding panels and PPI contributors? Exploring the impact of role descriptions, selection criteria, clear expectations, language, communication and handling conflict	96	0
1	TOPIC 29	PPI practices in selecting trial outcomes of importance to patients	A review of PPI practices that influence the primary outcomes within clinical trials, for example seizure control at 6 mo, time to healing. How often are these outcomes of importance to patients, and what role did PPI play in the decision‐making process?	96	0
1	TOPIC 31	A systematic review of PPI activity in improving the accessibility and usefulness of trial leaflets and information sheets for clinical trial participants	Patient/public contributors often help trial teams to design and produce information sheets. An assessment of existing research to evidence how PPI impacts patients understanding and acceptability of PIS within trials? How do PPI contributors write or review Patient Information Sheets? How often are they given guidance for this? Do trial teams listen to the advice of PPI contributors, how often are their changes adopted?	96	0
4	TOPIC 4	Adapting PPI to the particular needs of individual clinical trials	Research on how to tailor PPI plans to take into account key design features or specific patient groups, for example critically ill patients or children, including how the needs of clinical trials for PPI might change over the life of a trial. For example would a specific type of trial benefit from the use of patient panels rather than having one or two lay members on the trial steering committee?	92	0
4	TOPIC 9	The resources needed for PPI activity including time and money.	What are the resource implications for undertaking PPI? Do resource limitations impact upon PPI activity? What is spent on PPI activity for grant applications? How much budget is allocated within trials, what does it actually cost and is it possible to quantify the benefits in monetary terms? Evaluating current payment systems upon Involvement of PPI contributors at all stages of a trial	92	0
4	TOPIC 28	PPI practices to address the challenges of recruiting and retaining participants (eg patients) in clinical trials	Exploring the effectiveness of PPI practices to improve recruitment of patient participants (ie the people taking part as “subjects” in clinical trials), or help keep patients within a trial	92	0
7	TOPIC 30	PPI practices in selecting how to measure trial outcomes	A review of how PPI is used to decide on how outcomes are measured. For example how does PPI contribute to deciding whether a trial should collect data from patients using a weekly diary or a monthly questionnaire?	88	0
8	TOPIC 35	How is PPI involved in the dissemination of results and assessment of effectiveness?	A review of how PPI contributors are involved in writing lay reports for patient organizations or trial participants and presenting findings at conferences. Does involving PPI contributors impact on the effectiveness of dissemination? How often are funds available for this PPI work?	84	0
9	TOPIC 22	How do PPI contributors achieve and maintain an authentic patient perspective?	How does personal experience along with social demographics shape the perspective and input of a PPI contributor? Do PPI contributors become “professionalized” (ie more like researchers) over time? What helps to avoid this and keep them “in touch” with the authentic patient perspective? Do PPI contributors collect feedback from members of the public/other patients to help them in their role? If so what methods do they use and are they effective?	84	12
10	TOPIC 2	Effectiveness of different methods to capture wider patient or public perspectives on clinical trial designs, for example surveys, social media	PPI traditionally involves one person or small numbers of patients or public representatives seeking to share a “lay perspective” on trials. This research would look at ways to involve larger numbers of people in PPI within clinical trials.	80	0
10	TOPIC 33	What is the impact of PPI activity on the experience of patients who participate in a clinical trial?	Assessing the impact of PPI activity on a patients’ experience of trial participation, including their experience of consent, treatment, follow‐up and communication of the results	80	0

As discussed previously, an additional topic, regarding the definition of PPI and people's understanding of it, was added to round 2. Attendees gave low ratings for this topic, commenting that improved communication about the definition of PPI was needed within the trials community rather than more research on this definition. Of the six topics suggested by survey participants, only one (Topic 13: Exploring the role of PPI in the early stages of testing of new treatments [eg Phase 1 and Phase 2 trials]) reached consensus among meeting attendees (Table S3).

## 4 DISCUSSION

Through a consensus building process, we have identified priority topics for methodological research to inform PPI in clinical trials. The prioritized research topics were varied, covering PPI processes, resources, practices and relationships between stakeholder groups. The number and range of topics considered by more than 70% of meeting participants to be critically important indicates the high level of uncertainty and lack of evidence to inform PPI in clinical trials.^2^, ^4^, ^23^, ^27^ Meeting attendees were virtually unanimous about the most important PPI research priorities, with the top six achieving over 92% consensus.

Several of the top 10 prioritized research topics address concepts that are fundamental to PPI in clinical trials, such as productive working relationships, resources and how to adapt PPI models to avoid a one size fits all approach.^30^ Previous studies of PPI in clinical trials have particularly highlighted the importance of productive working relationships in creating the sort of environment to enable contributors to make a difference to research,^27^, ^40^, ^41^ whilst Barber et al., recommended considering PPI as a dynamic partnership rather than a procedural activity.^17^ During the consensus meeting many stakeholders shared examples of poor relationships between PPI contributors and researchers, also reflecting the high priority placed on the development of strong and productive partnerships between researchers and PPI contributors.

Whilst online resources such as INVOLVE provide costing tools for planning PPI, publications are poor at reporting the true costs.^42^ Topic 9 (resources needed for PPI activity), highlights uncertainties around PPI costs and points to concerns regarding the adequacy of funding to meet these costs. Research is, therefore, needed to help identify what level of resource is required for the implementation of PPI to ensure plans for such involvement are realistic and adequately supported. Whilst work is being undertaken to develop frameworks and guidelines to guide PPI practice in research,^43^, ^44^, ^45^ PPI plans and activities often vary according to context.^23^ Two of the top ten prioritized topics (Topics 4 and 2) point to concerns about current models of PPI,^29^, ^30^ highlighting the need for research to explore adaptations of PPI to the needs of particular trials, as well as methods to capture wider patient and public perspectives. For example, concerns were raised about current models of PPI being tokenistic, due to often small numbers (one or two) PPI contributors working on each trial.^10^, ^17^, ^18^ Research is needed to evaluate the effectiveness of different methods to increase diversity and capture wider patient or public perspectives on clinical trial designs, such as online surveys and social media.

Some of the top ten topics focus on the impact of PPI and particularly the need to review PPI in specific trial processes, such as: the development of trial information for patients; recruitment and retention of patients; choice and measurement of outcomes; and the dissemination of results. Two of these (Topic 28, strategies to recruit and retain patients, and Topic 29, the selection of trial outcomes) align with existing methodological research agendas for clinical trials.^38^ Conceptually, PPI should have a substantial role in addressing these issues. However, our results demonstrate that further work is needed to map and formally evaluate current PPI practices to help make these more relevant to trials,^3^, ^5^, ^6^, ^7^ and help to reduce research waste by targeting resources more effectively.^8^, ^9^ For example, it is common to involve patients in developing information materials for prospective trial participants,^43^ yet it is unclear whether or how this input increases participation rates or improves patient experience of research.^20^ A systematic review of PPI activity in the development of information materials for prospective trial participants (Topic 31) may provide evidence of the impact of such work, as well as inform future PPI in this important aspect of trial development.

During the consensus meeting some prioritized topics were revised to define a research method to be used to explore that particular topic, such as Topic 31: “A systematic review of PPI activity in improving accessibility and usefulness of trial leaflets and information sheets for clinical trial participants’*”*, whilst others, such as Topic 20 “Developing strong and productive working relationships between researchers and PPI contributors” are more wide ranging and relate to challenges in PPI. Such wider topics may contain multiple components, and further consideration will be needed to develop these topics into formal research questions and to identify the most appropriate research methods for addressing these questions.^32^

### 4.1 Strengths and limitations

Our study had several strengths. The METHODICAL team included representation of all stakeholder groups including lay and non‐lay members, who oversaw all stages of the project, including the recruitment strategy. The survey sample size was also relatively large compared to other Delphi studies, and attrition was low. Comparison of round 1 mean scores between those who did and did not complete round 2 indicate that our study was not affected by attrition bias (Figure S1).

We took several steps to help ensure that all stakeholder groups were represented at every stage of the Delphi and that all groups and individuals felt able to contribute freely. We sampled stakeholders purposively for the survey stage. For the consensus meeting a random selection of participants within groups ensured balance and fairness in the allocation places for lay and non‐lay stakeholders across all seven of stakeholder groups.

High and low priority topics identified in our study are cited in international literature on public and patient involvement in research.^46^, ^47^, ^48^ However, further research is required to explore the level of priority given to these topics in international settings.

The study also had some limitations. As the potential sample was large and diverse we were unable to fully define the sampling frame and used snowball sampling to try to make sure all stakeholder groups were included in the sample. As a result, our study was subject to self‐selection bias among those who registered for the study. To minimize the burden of survey participation, we chose not to collect social or demographic information, such as ethnicity or socio‐economic status; therefore, the diversity of participants and the potential impact of socio demographic characteristics upon the prioritization process cannot be evaluated.

Some study team members participated in the discussion and voting within the consensus meeting, which meant that a subset of attendees was not independent from the project. We reasoned that they would bring valuable experience and expertise to the discussion^49^ and therefore included them in the meeting. To promote transparency, at the beginning of the meeting all attendees introduced themselves and stated whether they took part in the survey, or whether they were a member of the study team. Care was taken in the facilitation of the meeting to ensure that all attendees had an equal opportunity to contribute to discussions. To help attendees feel free to vote as they wished during the meeting, voting was anonymous. Fifteen of 25 (60%) of attendees completed an optional feedback survey, of whom 14 of 15 (93%) were satisfied with how the meeting was facilitated and felt it produced a fair and independent outcome. However, as we did not track individual votes during the meeting, we are unable to present consensus meeting voting data by stakeholder group, or assess how individual scores differed from the online survey.

Delphis are dependent upon the participants having time to commit to the process to completion.^50^ To reduce the potential burden on participants and minimize attrition bias, we pre‐specified a two round, rather than a three or four round survey.^33^, ^51^, ^52^ While consensus was not achieved in the two round survey, it was achieved at the meeting, which highlights the value of face‐to‐face discussion and collective deliberation in reaching consensus.

Rather than beginning with an open question about possible topics and inviting suggestions from participants, the list of topics presented in round 1 was derived from the existing literature.^53^ However, we also invited participants to suggest additional topics in round 1. Despite a large number of suggested topics, relatively few new topics were suggested. Indeed, the majority of topics put forward by participants were already encompassed by existing topics. This perhaps indicates that our approach of presenting a list of topics in round 1 was an appropriate way of conducting a methodological research priority setting exercise in a context where not all stakeholders would be familiar with the concept of methodological research and might struggle to identify priorities without some examples as prompts.

## 5 CONCLUSIONS

In conclusion, the prioritized methodological research topics identified by the Delphi process highlight key uncertainties about PPI in trials. Addressing these uncertainties will be critical to enhancing PPI. Our findings should be used by those involved in planning and funding of PPI in clinical trials to help focus research efforts and minimize waste.

## CONFLICT OF INTEREST

All authors have completed the ICMJE uniform disclosure form and declare: no support from any organization for the submitted work; one author (JCC) receive funding from the NIHR Oxford Biomedical Research Centre to research the impact of PPI but did not participate in any of the consensus rounds, including the final consensus meeting so did not influence the outcome. No other authors have financial relationships with any organizations that might have an interest in the submitted work in the previous three years; no other relationships or activities that could appear to have influenced the submitted work.

## AUTHOR'S CONTRIBUTIONS

KW, CG, BY and PW conceived the study. KW led the study. PW oversaw statistical analyses. KW, AK, PW, BY, HB, CG, SD, DM, MC and TS designed the study. AK and KW wrote first draft of the manuscript. PW facilitated the consensus meeting. AK reviewed the literature to identify potential topics, organized the survey and consensus meeting and analysed the data under supervision from PW. All authors contributed to the study design, interpretation of data and revised and approved the final manuscript.

## ETHICAL APPROVAL

The IPHS Research Ethics Committee at the University of Liverpool reviewed and granted approval for this research on the 22nd September 2015 (Ref: IPHS‐1415‐VA‐212)
