# Supplementary material for: Priorities for methodological research on patient and public involvement in clinical trials: A modified Delphi process
Source: Health Expect. 2017 Jun 15;20(6):1401–10. doi: 10.1111/hex.12583 (PMC5689224; doi:10.1111/hex.12583)
Supplement: Supplementary file 1 [file HEX-20-1401-s001.docx]

**SUPPLEMENTARY FILE**

## Contents:

Table [S1: National networks approached to distribute survey invitation](#_Toc454267840)

Table S2: Description of research topics used within the Delphi process and a summary of the consensus meeting discussions

Table S3: The complete ranked list of methodological research priorities for PPI in clinical trials.

Table S4: Levels of consensus within stakeholder groups during the online Delphi

Figure S[5: Assessment of attrition bias during the online Delphi](#_Toc454267843)

##

## Table S1: National networks approached to distribute survey invitation

NIHR Clinical Research Network

MRC Hub for Trials Methodology Research Network

Trial Managers Network

HRA REC members

Wellcome Trust

Medical Research Council

UK Clinical Trial Research Collaboration’s network of registered Clinical Trial Units

CTU network of PPI coordinators

DSCHR

Welsh Government

RfPPB Wales

Health and Care Research Wales Public Involvement and Engagement Team

Involving People Network

Welsh Research Ethics Committees

NIHR Research Design Service members

## Table S2: Description of research topics used within the Delphi process and a summary of the consensus meeting discussions.

| **Research Topics and Categories (*Denotes topics added in round two)** | **Help Text (text changed during the meeting is in italics)** | **Summary of discussion at meeting** | **Summary of changes made at consensus meeting** |
| --- | --- | --- | --- |
| **Research on PPI practices and activity** | | | |
| 1. Developing common values, principles and standards for PPI specifically for clinical trials | Existing guidance from INVOLVE covers values, principles and standards for PPI across all types of health research. This work would develop a framework specifically for clinical trials, describing how PPI should be conducted within them. For example setting goals and objectives for PPI in a trial. | Discussion focussed upon whether clinical trials were distinct from other types of research involving PPI to warrant their own framework. It was suggested that the strong regulation and governance requirements of clinical trials made them unique and therefore should be considered separately. However, those who described the need for a specific framework for clinical trials did not necessarily feel it was a critical research priority. | None |
| 1. Effectiveness of different methods to capture wider patient or public perspectives on clinical trial designs e.g. surveys, social media | PPI traditionally involves one person or small numbers of patients or public representatives seeking to share a ‘lay perspective’ on trials. This research would look at ways to involve larger numbers of people in PPI within clinical trials. | Concerns were raised that the use of social media to involve larger numbers of people moved from involvement into engagement, which might dilute the concept of involvement. However, this wasn’t necessarily considered to be negative, as engagement, involvement and consultation are not mutually exclusive and can be mixed.  It was highlighted that voting it as critical was simply saying that more work needs to be done to understand this area and not that these are practices that everyone should be implementing. These queries and definitions could be explored within the research. | None |
| 1. Developing critical appraisal guidelines for funding boards to assess PPI activity within funding application forms | Clinical trialists are increasingly required to describe their PPI plans within funding applications, but how do funding boards assess this information? How feasible is the funding organisation’s PPI policy? Is research needed to develop guidelines for funding boards to help them assess the quality of PPI within clinical trial proposals? | There is variability in the assessment of PPI within funding boards. This topic was felt to be important although it could go further and include educating board members on the type of which would be feedback helpful to applicants. The counter argument was that whilst consistency is needed this is essentially a management job for grant panels and not a methodological research topic. | None |
| 1. Adapting PPI to the particular needs of individual clinical trials | Research on how to tailor PPI plans to take into account key design features or specific patient groups e.g. critically ill patients or children, including how the needs of clinical trials for PPI might change over the life of a trial, For example would a specific type of trial benefit from the use of patient panels rather than having one or two lay members on the trial steering committee? | Minimal discussion due to high level of agreement that this topic was important. | None |
| 1. Comparing the effectiveness of patient/public panels versus individual patients/members of the public in clinical trials | Research on which is more effective – involving panels (groups) of patients/public contributors within a trial, and/or one or two patient/public contributors? Is there an optimal number of PPI people for various activities? | It was considered important to look at the effectiveness of PPI, although concerns were raised that this topic simply picked out two methods for comparison from a wider group of methods, which could be covered within topic number 2. | None |
| 1. Defining the boundaries between PPI and qualitative research | Research on understanding what is PPI and what is qualitative research? Does there need to be a clear distinction to assist research teams and funding reviewers? | Attendees were reminded that this topic covered the definitions of qualitative research and PPI and did not include how quantitative research should be used within trials.  Discussion focussed upon the distinction between PPI and qualitative research and how qualitative techniques can be used to access a larger number and greater diversity of views during trial planning compared to one or two people sharing their views as PPI representative. However using qualitative research makes the person a research participant and not a PPI representative. | None |
| 1. Learning lessons from other academic sectors, public services, third sector and business to inform PPI models for clinical trials | Exploring PPI models from other sectors and types of health research to inform clinical trials. | Some attendees felt that exploring methods used by other sectors (e.g. education, charities, business) to encourage public engagement and participation would be worthwhile. However, others felt that whilst it was a good idea, it was not critically important. | None |
| 1. Mapping PPI activity and practices within UK Clinical Research Collaboration CTUs | Involvement of one of the 47 registered clinical trials units is often recommended to use their knowledge and expertise to ensure trials are well run and minimise resource waste. Research into how PPI conducted within this network, and what approaches are used to monitor and develop PPI activity. | Following a query about UK clinical trials units it was clarified that you do not need to use a registered clinical trials units to conduct a trial within the UK but some funders such as NIHR recommend they are approached and involved.  Discussion was minimal as this was not viewed to be a critically important topic | None |
| 1. The resources needed for PPI activity including time and money. | What are the resource implications for undertaking PPI? Do resource limitations impact upon PPI activity? What is spent on PPI activity for grant applications? How much budget is allocated within trials, what does it actually cost and is it possible to quantify the benefits in monetary terms? Evaluating current payment systems upon Involvement of PPI contributors at all stages of a trial. | Minimal discussion due to high level of agreement that this topic was important | None |
| 1. Assessing involvement of the wider trial team (e.g. statisticians, health economists) in planning and delivering PPI activity | Is PPI activity currently managed by Chief Investigators and trial managers/ trials co-ordinators? To what extent do other trial team members such as statisticians, data managers, qualitative methodologists and health economists help plan and implement PPI? Does who manages PPI activity impact upon trial design and conduct? | Minimal discussion due to high level of agreement that this topic was important | None |
| 1. Exploring the definition of PPI and people’s understanding of it* | What is PPI? What do different groups of people consider as PPI? What are the boundaries between involvement, engagement and participation in trials? (Whilst INVOLVE have written a definition of PPI, we have included this due to the number of people who took part in round 1 of this Delphi who suggested it as a new topic) | Attendees discussed that there was no need for research just better communication of the definition of PPI written by INVOLVE. | None |
| 1. Understanding how Research Ethics Committees review PPI plans and activity in trials* | How are PPI plans and activity in the design process of a trial taken into account during the ethics review process? Does PPI affect the ethical decision making process? Do different ethics committees take similar approaches to reviewing PPI? | It was highlighted that the Health Research Authority (HRA) are working to address this. However, it was felt that ongoing work shouldn’t influence voting. Instead a note in the report would be made to inform people that this work is being conducted by the HRA. | None |
| 1. Exploring the role of PPI in the early stages of testing of new treatments (e.g. Phase 1 and Phase 2 trials)* | Understanding current PPI practices in Phase 1 trials (where new treatments are tested in a small number of *patients*) and in Phase 2 trials (where new treatments are tested in a larger group of *patients* with the same health condition). Identifying how PPI can be developed in early phase trials. *This topic does not include Phase 1 trials that test interventions in healthy volunteers* | Discussion focussed on the need for research to understand the circumstances where PPI is important in early stage (Phase 1 and 2) trials. In these trials patients can be exposed to an intensive range of investigations, which may not all be absolutely needed, or may be questioned with PPI.  A distinction was made between early phase one trials that included patients (for example in cancer trials) and those testing new interventions on healthy volunteers. | References to healthy participants were removed to focus on patients enrolling in phase 1 and 2 trials. |
| 1. To what extent are lay members involved in reviewing manuscripts for journals and what is the impact of this?* | How are patients and members of the public involved in reviewing articles for publication within journals? What influence do they have within the editorial process? How does this work alongside or as part of the peer (professional) review process? | Minimal discussion due to high level of agreement that this topic was important. | None |
| **Research on finding and selecting PPI contributors** | | | |
| 1. Sources for identifying and approaching PPI contributors e.g. social media, charities, community organisation. | Research on how are PPI contributors are identified and approached to become involved in the design and conduct of clinical trials? What are the most effective sources/ways to do this and what are the challenges? | Minimal discussion due to high level of agreement that this topic was important. | None |
| 1. Assessment of different methods (e.g. social media, incentives) to increase the diversity of PPI contributors (age, ethnicity, socioeconomic status, disability) | Research on how diverse PPI contributors are within trials? Is there good inclusion of people of different ages, ethnicities, socioeconomic, educational backgrounds? What groups are not currently involved and what methods can ensure people from a wider range of different backgrounds and walks of life get involved? | The similarities between topics numbers 16, 17 and 18 were highlighted and discussed. However it was clarified this topic is specifically focused on research to inform how best to increase diversity of PPI contributors. | None |
| 1. Reasons for involvement and refusing involvement by potential PPI contributors | Research on why patients and members of the public become involved within trials and why might they decline invitations? Why might people feel they have nothing to contribute? | It was clarified that this topic is focused on the reasons why patients may or may not want to be involved in the design and oversight of a trial (acting as an advisor rather than a trial participant).  An overlap with topic number 19 was raised but that it was suggested that 19 covered the next step in the PPI process, after patients and members of the public have said yes to an advisory role. | None |
| 1. Selection methods for PPI contributors | Research on what criteria (e.g. PPI person specifications) and processes (interviews) are used to select PPI contributors? Do trial teams tend to select people who share their views, or are already known to them and if so how does this influence the effectiveness of PPI? | Concerns were discussed about The EPIC study finding that many doctors were inviting patients already known to them. It was felt this could impact PPI quality if a patient’s contributions were influenced by concerns about affecting the relationship with their healthcare provider.  It was clarified that this topic is about how PPI contributors are selected or chosen for PPI roles. | None |

| **Research on implementing PPI** | | | |
| --- | --- | --- | --- |
| 1. Review of factors influencing the attendance of PPI contributors at Trial steering committee and Trial Management group meetings | Exploration of the attendance levels by PPI contributors, along with any effective approaches which could maximise attendance? For example does the approach to organising meetings limit PPI contributors attendance? What options are available for maintaining the involvement of people with poor health status? | Concerns were raised about focus being placed on the attendance of PPI contributors at trial steering committees, compared to how they contribute whilst they are there. However, it was also described how the EPIC survey found that face to face meetings allowed better relationships to form between contributors and investigators, although it was often hard for contributors to attend meetings.  It was clarified that topic was about research to look at the PPI contributor’s involvement specifically at meetings once they have agreed to be involved. | None |
| 1. Developing strong and productive working relationships between researchers and PPI contributors | Research on what defines and enables a good working relationship between researchers on a trial team, trial committee (e.g. trial steering committee or ethics committee) or funding panels and PPI contributors? Exploring the impact of role descriptions, selection criteria, clear expectations, language, communication and handling conflict. | Examples of problems in relationships between PPI contributors and researchers were discussed. One person commented that they work with several groups who have no appreciation of PPI and therefore they thought this topic was of critical importance. | None |
| 1. Exploring why PPI contributors may want or need to stop their involvement early. | Clinical trials can run for many years, making it difficult for people to be involved throughout the lifetime of a trial. This research would explore the reasons why PPI contributors may need or want to stop their involvement, along with the benefits of PPI contributor continuity and practices that help facilitate longer term involvement. | It was clarified that this topic is broad and covers why people stop any kind of involvement (in comparison to topic number 19, which is specifically focused on meetings).  It was also raised that there could be a link between this topic and topic number 20. | None |
| 1. How do PPI contributors achieve and maintain an authentic patient perspective? | How does personal experience along with social demographics shape the perspective and input of a PPI contributor? Do PPI contributors become “professionalised” (i.e. more like researchers) over time? What helps to avoid this and keep them “in touch” with the authentic patient perspective? Do PPI contributors collect feedback from members of the public/ other patients to help them in their role? If so what methods do they use and are they effective? | Comments were raised about whether you could ever describe anyone as an ‘inauthentic patient’. It was also unclear whether PPI representatives can become professionalised.  It was suggested that this is an ongoing debate and therefore the topic has been included to help decide whether it is a priority for research. | None |
| **Research on training, induction and support** | | | |
| 1. Training needs assessment for researchers, trial managers and PPI contributors | Do PPI contributors, chief investigators, trial managers and other trial staff want or need training on PPI in clinical trials? What training is currently available and what needs developing? | It was clarified that that this topic is about assessing training needs not identifying why people don’t want to engage with training.  It was suggested that plenty of research has shown the importance of training and so this a communication issue rather than an idea for future research. People need to encourage their peers apply the lessons learnt and engage with training. | None |
| 1. Ongoing support and development for PPI contributors | How are PPI contributors supported throughout their roles? Do PPI contributors with physical difficulties (e.g. hearing loss or mobility limitations) receive appropriate support to assist engagement? Do PPI contributors want opportunities for feedback and development within their roles? | Overlap with other topics in this section were discussed. It was felt that training and support were highly important but it could be difficult to consider them individually.  It was clarified this topic was specifically looking at what research could be done to inform how to support people in their roles as PPI contributors and whether existing support arrangements are appropriate. | None |
| 1. Ways to *negotiate with* PPI contributors about their role within a trial (induction practices) | Exploring the content and format of effective ways to help PPI contributors learn about their roles in trials and refine or *negotiate* their roles. How are training needs assessed at induction? | People shared personal experiences of weaknesses within induction practices and agreed that this topic was important. However there was a discussion around the role of research compared to governance.  The EPIC (Evidence base for Patient and public Involvement in Clinical trials) investigated PPI in a cohort of trials funded by the NIHR Health Technology Assessment (HTA) programme. It was described how the results of this study identified a mismatch between researchers’ expectations and how PPI contributors perceive they can contribute. There is a need to help people understand how they contribute across a broad range of roles. For example, helping PPI contributors understand how they can be involved in the analysis of a trial even though they don’t have technical knowledge. | The word inform was removed from the topic title and replaced with ‘negotiate with’ to emphasise the two way process. |
| 1. Assessing the impact of PPI activity on PPI contributors. | What makes for a positive vs. negative experience of involvement? What is the impact on PPI contributors’ skills, knowledge, self-esteem, time, emotional burden, career opportunities, etc.? | Attendees discussed how important this topic was compared to other topics in the survey. It was raised that there are a lot of research publications on this topic compared to other topics in the survey. Although it was noted that the proportion of evidence specifically relating to clinical trials is not known.  As research for this topic is published, it was agreed that this should be taken into account during voting for this topic. | None |
| 1. Assessing the impact of PPI activity on trial staff | What makes for a positive vs. negative experience of PPI? What is the impact on trial staff time, productivity, skills, confidence, motivation, perceived power, career etc? | Attendees felt that not all Chief Investigators are signed up to PPI and there is a lot of tokenism and misunderstanding so research on this topic is needed to understand their concerns. There doesn’t seem to be much work on how PPI impacts on trial staff, and in particular, what affect this has on future trials undertaken by the Chief Investigators. | None |

| **Research to develop an evidence base for effective practice** | | | |
| --- | --- | --- | --- |
| 1. PPI practices to address the challenges of recruiting and retaining participants (e.g. patients) in clinical trials | Exploring the effectiveness of PPI practices to improve recruitment of patient participants (i.e. the people taking part as ‘subjects’ in clinical trials), or help keep patients within a trial. | Minimal discussion due to high level of agreement that this topic was important. | None |
| 1. PPI practices in selecting trial outcomes of importance to patients | A review of PPI practices that influence the primary outcomes within clinical trials e.g. seizure control at 6 months, time to healing. How often are these outcomes that are important to patients, and what role did PPI play in the decision making process? | Minimal discussion due to high level of agreement that this topic was important. | None |
| 1. PPI practices in selecting how to measure trial outcomes | A review of how PPI is used to decide on how outcomes are measured. For example how does PPI contribute to deciding whether a trial should collect data from patients using a weekly diary or a monthly questionnaire? | It was noted that continued data collection during a trial can be a burden for patients. PPI could be used to identify when data collection could be causing a burden and potentially result in patients leaving a trial. | None |
| 1. *A systematic review* of PPI activity in improving the accessibility and usefulness of trial leaflets and information sheets for clinical trial participants | Patient/public contributors often help trial teams to design and produce information sheets*. An assessment of existing research* to *evidence how PPI impacts patients understanding and acceptability of PIS within trials*? How do PPI contributors write or review Patient Information Sheets? How often are they given guidance for this? Do trial teams listen to the advice of PPI contributors, how often are their changes adopted? | It was discussed how there is evidence that Patient Information leaflets (PIS) leaflets written by PPI contributors / patients are better received than those that aren’t. However, there is no real evidence about how patients should be included in this process. The need for a systematic review rather than new research was discussed. | The topic title and help text was amended to emphasise the need for a systematic review of published research rather than new investigations. |
| 1. Reviewing PPI *and the involvement of patients* in setting research priorities for different health conditions | A review of how PPI *and patients* *are involved* in choosing what research is undertaken within clinical trials. | It was discussed how the James Lind Alliance (JLA) carries out a lot of this work, although the approach they use is not always the same. Other groups carry out similar work to the JLA.  A change to the topic title to include to ‘the involvement of patients’ was recommended. | Both the title and help text were expanded to include reference to ‘the involvement of patients’ alongside PPI in setting research priorities. |
| 1. What is the impact of PPI activity on the experience of patients who participate in a clinical trial? | Assessing the impact of PPI activity on a patients experience of trial participation, including their experience of consent, treatment, follow up and communication of the results. | It was discussed how research into this topic was important in order to provide robust evidence about how important PPI is in improving research participant’s experience of clinical trials. | None |
| 1. Review of PPI practices in trials that stopped early | Are there any lessons that can be learnt about PPI from trials that stopped early *for problems associated with trial design and conduct e.g. poor recruitment or retention*? For example, reviewing the reasons for stopping the trial early, trial context and PPI activity? *This does not include trials that stopped early due to treatment effect (e.g. confirmed benefit or suspected harm)* | Discussion centred on the reasons why a trial might stop early. Not all of these reasons might be related to PPI for example if a trial is stopped for benefit, such as when a treatment effect has been identified early.  In general attendees frequently interpreted the question as focusing on investigating trials that stopped early due to poor recruitment or patient issues rather than positive treatment related reasons. | Help text was amended to focus the research on trials that stopped early due to patient centred reasons compared to statistical reasons. E.g. recruitment or retention difficulties rather than stopping a trial for benefit |
| 1. How is PPI involved in the dissemination of results and assessment of effectiveness? | A review of how PPI contributors are involved in writing *lay reports* for patient organisations or trial participants and presenting findings at conferences. Does involving PPI contributors impact on the effectiveness of dissemination? How often are funds available for this PPI work? | It was felt this topic was important to build partnership between PPI contributors and researchers. However, research should also consider how PPI is involved in ensuring trial results are delivered sensitively to participants.  It was suggested that the help text should be revised as lay summary are written at the beginning of a trial rather than during dissemination of results. | Text was amended to correct terminology. ‘Lay summaries’ was replaced by ‘lay reports’. |
| 1. Funding application timescales and the impact on PPI quality in designing clinical trials | *What is the impact* of timescales for preparing and submitting funding applications impact on the plans *and does this affect the* quality of PPI in the early stages of a trial? *What is the best application of PPI within the possible timeframes?* | Many applications do not involve PPI at the design stage and this is perceived to be a problem with the current UK funding process. It would be helpful if funders were more specific in the brief about the type of PPI that they would expect within the timescales.  There was a mixed opinion on whether research was the solution despite agreement that it is a problem. However, some felt that whilst it was an assumption that short timescales are leading to poor PPI and that evidence is needed to prove this and create change in practice. | Help text was changed from assessing ‘how short funding timescales impact on PPI quality’ to exploring the effect of timescales and the best use of PPI within the time available to avoid assumptions of impact. |
| 1. Core outcomes for assessing PPI impact | Development of a minimum set of outcomes for assessing PPI impact and agreement about how these should be measured. A set of core outcomes for PPI would allow comparison across trials, however it is unclear what is most important to assess. For example PPI impact could be measured on recruitment rates, retention rates, levels of missing data, or need for funding extension. | People queried the feasibility of this topic at the present time due to variety of trial designs and the ability to separate key comparable topics. However this didn’t mean it wasn’t important. Core outcomes would enable people to assess and compare PPI across trials which would be very important.  NB: Following voting on this topic it was discussed how topic 37 should be explored prior to topic 38, as what outcomes you measure need to be established first. The wording of the topic was amended to reflect this |  |
| 1. What methods should we use to measure the impact of PPI?* | What methods should be used to assess PPI impact? How do we measure both positive and negative impacts and what factors should be considered when measuring impact e.g. type of PPI contribution? Who should make the assessment of impact, e.g. researchers, patients or both? | No vote was taken (See above) | . |
| 1. What activities or characteristics of PPI lead to a successful trial?* | How do different PPI activities and/or different ways of involving patients and members of the public contribute to different levels of trial success? What is the impact of ‘Professional’ PPI representatives, patients, service users and other examples of PPI on trial success? What is the impact of PPI funding? How do we ensure continuous improvement in the quality of PPI to increase the likelihood of trial success? | It was felt this was an important issue but that it was too general and covered in other questions. Some attendees raised questions about how you define a ‘successful trial’ No vote performed, topic removed due to overlap. |  |
| **Research on reporting PPI activity and Impact** | | | |
| 1. The inclusion of PPI activity within existing trial guidelines e.g. SPIRIT, CONSORT | Guidelines inform the practices and processes within Clinical Trials. For example SPIRIT offers a framework for writing trial protocols and CONSORT for publishing trial results. Do existing guidelines include recommendations around PPI, and what *if anything* should be included? | Discussion focussed on whether this topic is talking about reporting PPI in separate publications or reporting it within trial reports published in journals.  There were mixed views on the value of reporting PPI activity within trial reports based on the restrictive word counts and whether reports should, at a bare minimum, include that PPI was undertaken. Whilst topic numbers 40 and 42 were considered similar, it was felt that reporting PPI within both trial reports and separate PPI publications was of value. This topic was specifically focused on researching whether there is agreement that PPI should be included within the guidelines and documents such as CONSORT. | The words ‘if anything’ added to show that there may not be value in adding PPI to these guidelines. |
| 1. Do journals review PPI activity during the editorial process | When considering a trial report for publication, do journal editors consider or assess PPI? Do they give any feedback about PPI reporting? | It was felt that most journals are unlikely to review PPI activity as it is not recommended within CONSORT and so would be difficult to encourage journals to adopt this.  It was discussed whether the question should be broadened to consider whether journals ‘should’ review PPI? However, this seemed too broad and the process of reviewing of PPI in journals was not linked to an outcome so lacked value to several people. As a result no amendments were made to the topic text prior to voting. | None |
| 1. Guidance for reporting PPI activity and impact in trial publications | How frequently is PPI activity included within journal articles and newspaper reports of clinical trials? What type of information is communicated and is it more frequently reported in certain journals or for certain types of trials? How should PPI activity be reported given the restrictive word counts within journals*? When should PPI be reported in main trial publications and when should a separate PPI focused paper or hybrid approach be used?* What terms would be useful for tagging PPI research within databases of journal articles (e.g. MESh terms) to allow researchers to identify and use data across trials? | Following on from discussions within topic number 40, it was felt this topic got to the heart of the issue as to whether PPI should be reported in trial publications. It was suggested that the topic should include guidance on how PPI should be reported for example when to create a separate publication, when it should be included in the trial report and when a hybrid approach is desirable. | Help text was amended to emphasise the need to explore when PPI should be reported in main trial publications and when a separate publication or hybrid approach is preferable. |

## Table S3: The complete ranked list of methodological research priorities for PPI in clinical trials.

| Ranking | Topic No. | **Topic Title** | **Number of scores 1-9^a^** | **Percentage of scores 7-9** | **Percentage of scores 1-3** |
| --- | --- | --- | --- | --- | --- |
| 1 | TOPIC 20 | Developing strong and productive working relationships between researchers and PPI contributors | 25 | 96% | 0% |
| 1 | TOPIC 29 | PPI practices in selecting trial outcomes of importance to patients | 25 | 96% | 0% |
| 1 | TOPIC 31 | A systematic review of PPI activity in improving the accessibility and usefulness of trial leaflets and information sheets for clinical trial participants | 25 | 96% | 0% |
| 4 | TOPIC 4 | Adapting PPI to the particular needs of individual clinical trials | 25 | 92% | 0% |
| 4 | TOPIC 9 | The resources needed for PPI activity including time and money. | 25 | 92% | 0% |
| 4 | TOPIC 28 | PPI practices to address the challenges of recruiting and retaining participants (e.g. patients) in clinical trials | 25 | 92% | 0% |
| 7 | TOPIC 30 | PPI practices in selecting how to measure trial outcomes | 25 | 88% | 0% |
| 8 | TOPIC 35 | How is PPI involved in the dissemination of results and assessment of effectiveness? | 25 | 84% | 0% |
| 9 | TOPIC 22 | How do PPI contributors achieve and maintain an authentic patient perspective? | 25 | 84% | 12% |
| 10 | TOPIC 2 | Effectiveness of different methods to capture wider patient or public perspectives on clinical trial designs e.g. surveys, social media | 25 | 80% | 0% |
| 10 | TOPIC 33 | What is the impact of PPI activity on the experience of patients who participate in a clinical trial? | 25 | 80% | 0% |
| 12 | TOPIC 3 | Developing critical appraisal guidelines for funding boards to assess PPI activity within funding application forms | 24 | 79% | 0% |
| 13 | TOPIC 13^b^ | Exploring the role of PPI in the early stages of testing of new treatments (e.g. Phase 1 and Phase 2 trials) | 24 | 79% | 4% |
| 14 | TOPIC 32 | Reviewing PPI and the involvement of patients in setting research priorities for different health conditions. | 25 | 76% | 0% |
| 15 | TOPIC 37 | Core outcomes for assessing PPI impact | 25 | 76% | 4% |
| 16 | TOPIC 16 | Assessment of different methods (e.g. social media, incentives) to increase the diversity of PPI contributors (age, ethnicity, socioeconomic status, disability) | 25 | 72% | 4% |
| 17 | TOPIC 24 | Ongoing support and development for PPI contributors | 25 | 68% | 0% |
| 18 | TOPIC 25 | Ways to negotiate PPI contributors about their role within a trial (induction practices) | 25 | 64% | 4% |
| 19 | TOPIC 40 | The inclusion of PPI activity within existing trial guidelines e.g. SPIRIT, CONSORT | 25 | 64% | 8% |
| 20 | TOPIC 27 | Assessing the impact of PPI activity on trial staff | 24 | 63% | 4% |
| 21 | TOPIC 1 | Developing common values, principles and standards for PPI specifically for clinical trials | 25 | 60% | 8% |
| 22 | TOPIC 34 | Review of PPI practices in trials that stopped early | 25 | 60% | 12% |
| 23 | TOPIC 42 | Guidance for reporting PPI activity and impact in trial publications | 24 | 54% | 13% |
| 24 | TOPIC 36 | Funding application timescales and the impact on PPI quality in designing clinical trials | 25 | 52% | 4% |
| 25 | TOPIC 12^b^ | Understanding how Research Ethics Committees review PPI plans and activity in trials | 25 | 48% | 12% |
| 26 | TOPIC 23 | Training needs assessment for researchers, trial managers and PPI contributors | 25 | 44% | 12% |
| 27 | TOPIC 19 | Review of factors influencing the attendance of PPI contributors at Trial steering committee and Trial Management group meetings | 25 | 44% | 16% |
| 28 | TOPIC 18 | Selection methods for PPI contributors | 25 | 44% | 20% |
| 29 | TOPIC 7 | Learning lessons from other academic sectors, public services, third sector and business to inform PPI models for clinical trials | 25 | 40% | 12% |
| 30 | TOPIC 26 | Assessing the impact of PPI activity on PPI contributors. | 24 | 38% | 8% |
| 31 | TOPIC 17 | Reasons for involvement and refusing involvement by potential PPI contributors | 25 | 36% | 12% |
| 32 | TOPIC 15 | Sources for identifying and approaching PPI contributors e.g. social media, charities, community organisation. | 25 | 24% | 4% |
| 33 | TOPIC 21 | Exploring why PPI contributors may want or need to stop their involvement early. | 25 | 24% | 24% |
| 34 | TOPIC 41 | Do journals review PPI activity during the editorial process | 24 | 17% | 42% |
| 35 | TOPIC 14^b^ | To what extent are lay members involved in reviewing manuscripts for journals and what is the impact of this? | 25 | 16% | 48% |
| 36 | TOPIC 5 | Comparing the effectiveness of patient/public panels versus individual patients/members of the public in clinical trials | 25 | 16% | 56% |
| 37 | TOPIC 6 | Defining the boundaries between PPI and qualitative research | 24 | 13% | 54% |
| 38 | TOPIC 10 | Assessing involvement of the wider trial team (e.g. statisticians, health economists) in planning and delivering PPI activity | 25 | 12% | 36% |
| 39 | TOPIC 11^b^ | Exploring the definition of PPI and people’s understanding of it | 25 | 12% | 36% |
| 40 | TOPIC 8 | Mapping PPI activity and practices within UK Clinical Research Collaboration CTUs | 24 | 8% | 21% |
| - | TOPIC 38^bc^ | What methods should we use to measure the impact of PPI? | - | - | - |
| - | TOPIC 39^bc^ | What activities or characteristics of PPI lead to a successful trial? | - | - | - |

**Legend:** Topics are numbered in the order presented within the survey. ^a^Meeting attendees could abstain from scoring a topic by choosing a score of 10. ^b^These six topics were suggested by participants and added in round 2. ^c^METHODICAL meeting attendees felt that these topics overlapped with other topics and so no voting was undertaken.

## Table S4: Levels of agreement within stakeholder groups during the online Delphi

**Notes:** The topic number represents the order in which the topics were presented in both rounds of the survey. Cells are coloured dark grey to indicate that 70% or more of each stakeholder group agreed that the topic was critically important (scored 7-9). Cells are coloured light grey if 51-69.99% of stakeholders scored the topic 7-9. *Full consensus was not achieved as over 15% of stakeholders also scored it 1-3.

| **Final Ranking** | **Included In Round 1** | **Topic Number** | **Final Title** | **Percentage of stakeholders scoring 7-9** | | | | | | | | | | | | | |
| --- | --- | --- | --- | --- | --- | --- | --- | --- | --- | --- | --- | --- | --- | --- | --- | --- | --- |
|  |  |  |  | **Round 1** | | | | | | | **Round 2** | | | | | | |
|  |  |  |  | **Lay reviewers** | **Non-Lay reviewers** | **PPI Planners** | **PPI Advisors** | **PPI Contributors** | **PPI Coordinators** | **PPI Researchers** | **Lay reviewers** | **Non-Lay reviewers** | **PPI Planners** | **PPI Advisors** | **PPI Contributors** | **PPI Coordinators** | **PPI Researchers** |
| 1 | y | 20 | Developing strong and productive working relationships between researchers and PPI contributors | 87% | 38% | 58% | 58% | 89% | 92% | 75% | 77% | 44% | 87% | 67% | 100% | 92% | 75% |
| 1 | y | 31 | A systematic review of PPI activity in improving the accessibility and usefulness of trial leaflets and information sheets for clinical trial participants | 74% | 49% | 58% | 58% | 66% | 80% | 46% | 90% | 52% | 82% | 58% | 81% | 84% | 42% |
| 1 | y | 29 | PPI practices in selecting trial outcomes of importance to patients | 70% | 63% | 67% | 67% | 74% | 88% | 69% | 74% | 64% | 79% | 92% | 85% | 88% | 83% |
| 4 | y | 4 | Adapting PPI to the particular needs of individual clinical trials | 64% | 61% | 67% | 67% | 58% | 60% | 92% | 68% | 67% | 62% | 75% | 74% | 76% | 92% |
| 4 | y | 28 | PPI practices to address the challenges of recruiting and retaining participants (e.g. patients) in clinical trials | 66% | 50% | 75% | 75% | 75% | 92% | 54% | 74% | 61% | 79% | 83% | 81% | 92% | 75% |
| 4 | y | 9 | The resources needed for PPI activity including time and money. | 71% | 50% | 67% | 67% | 74% | 74% | 85% | 79% | 53% | 67% | 67% | 89% | 76% | 92% |
| 7 | y | 30 | PPI practices in selecting how to measure trial outcomes | 64% | 42% | 50% | 50% | 53% | 71% | 69% | 74% | 42% | 56% | 50% | 70% | 83% | 67% |
| 8 | y | 35 | How is PPI involved in the dissemination of results and assessment of effectiveness? | 57% | 42% | 58% | 58% | 61% | 80% | 77% | 67% | 45% | 62% | 50% | 73% | 80% | 67% |
| 9 | y | 22 | How do PPI contributors achieve and maintain an authentic patient perspective? | 80% | 50% | 67% | 67% | 64% | 79% | 82% | 74% | 61% | 77% | 75% | 81% | 88% | 82% |
| 10 | y | 2 | Effectiveness of different methods to capture wider patient or public perspectives on clinical trial designs e.g. surveys, social media | 53% | 53% | 42% | 42% | 47% | 72% | 85% | 53% | 48% | 69% | 33% | 63% | 76% | 75% |
| 10 | y | 33 | What is the impact of PPI activity on the experience of patients who participate in a clinical trial? | 59% | 53% | 50% | 50% | 66% | 71% | 69% | 74% | 64% | 51% | 50% | 70% | 80% | 67% |
| 12 | y | 3 | Developing critical appraisal guidelines for funding boards to assess PPI activity within funding application forms | 60% | 53% | 50% | 50% | 54% | 91% | 54% | 55% | 58% | 62% | 67% | 67% | 96% | 67% |
| 13 | n | 13 | Exploring the role of PPI in the early stages of testing of new treatments (e.g. Phase 1 and Phase 2 trials) | - | - | - | - | - | - | - | 54% | 33% | 24% | 33% | 59% | 68% | 50% |
| 14 | y | 32 | Reviewing PPI and the involvement of patients in setting research priorities for different health conditions | 73% | 49% | 67% | 67% | 77% | 75% | 75% | 77% | 53% | 64% | 67% | 93% | 83% | 75% |
| 15 | y | 37 | Core outcomes for assessing PPI impact | 62% | 42% | 50% | 50% | 71% | 86% | 54% | 67% | 48% | 64% | 58% | 65% | 83% | 50% |
| 16 | y | 16 | Assessment of different methods (e.g. social media, incentives) to increase the diversity of PPI contributors (age, ethnicity, socioeconomic status, disability) | 71% | 50% | 42% | 42% | 58% | 92% | 54% | 68% | 55% | 69% | 58% | 67% | 92% | 58% |
| 17 | y | 24 | Ongoing support and development for PPI contributors | 58% | 45% | 75% | 75% | 64% | 80% | 54% | 62% | 42% | 59% | 75% | 78% | 76% | 67% |
| 18 | y | 25 | Ways to negotiate with PPI contributors about their role within a trial (induction practices) | 65% | 47% | 58% | 58% | 51% | 64% | 58% | 67% | 48% | 54% | 58% | 74% | 60% | 75% |
| 19 | y | 40 | The inclusion of PPI activity within existing trial guidelines e.g. SPIRIT, CONSORT | 66% | 34% | 50% | 50% | 68% | 76% | 54% | 56% | 33% | 41% | 58% | 69% | 77% | 67% |
| 20 | y | 27 | Assessing the impact of PPI activity on trial staff | 59% | 39% | 67% | 67% | 58% | 60% | 62% | 55% | 36% | 33% | 50% | 63% | 60% | 50% |
| 21 | y | 1 | Developing common values, principles and standards for PPI specifically for clinical trials | 64% | 50% | 58% | 58% | 58% | 72% | 67% | 67% | 61% | 56% | 58% | 56% | 76% | 64% |
| 22 | y | 34 | Review of PPI practices in trials that stopped early | 51% | 34% | 67% | 67% | 53% | 60% | 69% | 50% | 45% | 41% | 67% | 63% | 72% | 67% |
| 23 | y | 42 | Guidance for reporting PPI activity and impact in trial publications | 53% | 18% | 33% | 33% | 57% | 57% | 38% | 51% | 24% | 54% | 33% | 65% | 57% | 50% |
| 24 | y | 36 | Funding application timescales and the impact on PPI quality in designing clinical trials | 55% | 38% | 50% | 50% | 47% | 70% | 67% | 56% | 30% | 51% | 42% | 52% | 70% | 55% |
| 25 | n | 12 | Understanding how Research Ethics Committees review PPI plans and activity in trials | - | - | - | - | - | - | - | 56% | 33% | 38% | 50% | 41% | 48% | 42% |
| 26 | y | 23 | Training needs assessment for researchers, trial managers and PPI contributors | 57% | 37% | 67% | 67% | 59% | 76% | 69% | 64% | 30% | 67% | 67% | 69% | 80% | 75% |
| 27 | y | 19 | Review of factors influencing the attendance of PPI contributors at Trial steering committee and Trial Management group meetings | 36% | 24% | 42% | 42% | 47% | 46% | 62% | 33% | 30% | 34% | 33% | 63% | 44% | 50% |
| 28 | y | 18 | Selection methods for PPI contributors | 57% | 42% | 33% | 33% | 53% | 44% | 69% | 64% | 42% | 46% | 42% | 59% | 48% | 67% |
| 29 | y | 7 | Learning lessons from other academic sectors, public services, third sector and business to inform PPI models for clinical trials | 41% | 29% | 17% | 17% | 26% | 63% | 50% | 38% | 24% | 26% | 8% | 22% | 58% | 50% |
| 30 | y | 26 | Assessing the impact of PPI activity on PPI contributors. | 52% | 32% | 33% | 33% | 47% | 64% | 58% | 59% | 34% | 39% | 25% | 44% | 72% | 67% |
| 31 | y | 17 | Reasons for involvement and refusing involvement by potential PPI contributors | 54% | 37% | 33% | 33% | 61% | 56% | 46% | 59% | 33% | 46% | 42% | 63% | 52% | 42% |
| 32 | y | 15 | Sources for identifying and approaching PPI contributors e.g. social media, charities, community organisation. | 61% | 50% | 50% | 50% | 60% | 72% | 54% | 59% | 52% | 56% | 50% | 62% | 68% | 50% |
| 33 | y | 21 | Exploring why PPI contributors may want or need to stop their involvement early. | 40% | 26% | 42% | 42% | 36% | 54% | 46% | 41% | 24% | 23% | 25% | 44% | 48% | 50% |
| 34 | y | 41 | Do journals review PPI activity during the editorial process | 45% | 19% | 25% | 25% | 57% | 48% | 31% | 47% | 16% | 18% | 33% | 54% | 48% | 42% |
| 35 | n | 14 | To what extent are lay members involved in reviewing manuscripts for journals and what is the impact of this? | - | - | - | - | - | - | - | 35% | 10% | 8% | 17% | 44% | 28% | 42% |
| 36 | y | 5 | Comparing the effectiveness of patient/public panels versus individual patients/members of the public in clinical trials | 38% | 39% | 33% | 33% | 33% | 33% | 42% | 37% | 21% | 31% | 33% | 22% | 32% | 17% |
| 37 | y | 6 | Defining the boundaries between PPI and qualitative research | 40% | 32% | 50% | 50% | 19% | 30% | 42% | 37% | 34% | 26% | 42% | 15% | 33% | 50% |
| 38 | y | 10 | Assessing involvement of the wider trial team (e.g. statisticians, health economists) in planning and delivering PPI activity | 45% | 21% | 25% | 25% | 41% | 46% | 31% | 39% | 9% | 15% | 17% | 30% | 46% | 17% |
| 39 | n | 11 | Exploring the definition of PPI and people’s understanding of it | - | - | - | - | - | - | - | 61% | 39% | 33% | 50% | 52% | 52% | 33% |
| 40 | y | 8 | Mapping PPI activity and practices within UK Clinical Research Collaboration CTUs | 58% | 24% | 42% | 42% | 47% | 52% | 46% | 47% | 15% | 28% | 33% | 44% | 48% | 42% |
| - | n | 39 | What activities or characteristics of PPI lead to a successful trial? | - | - | - | - | - | - | - | 76% | 61% | 77% | 50% | 77% | 84% | 83%* |
| - | n | 38 | What methods should we use to measure the impact of PPI? | - | - | - | - | - | - | - | 68% | 66% | 77% | 50% | 76% | 76% | 67% |

## Figure S1: Analysis of attrition bias during the online Delphi

Mean scores from round one across all research topics for participants who:

Completed round two

Dropped out before round two

| 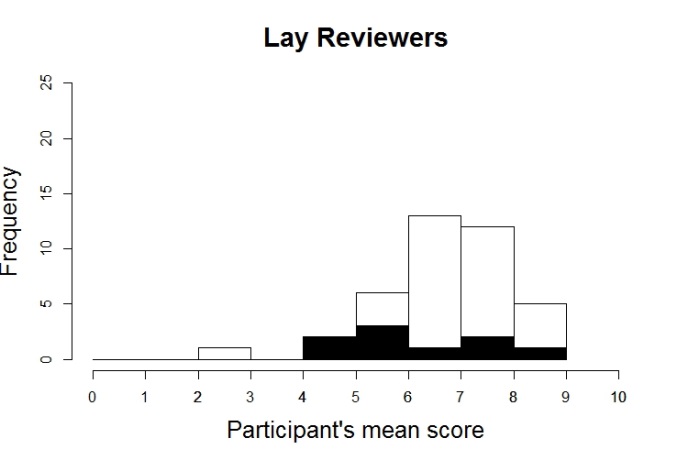 | 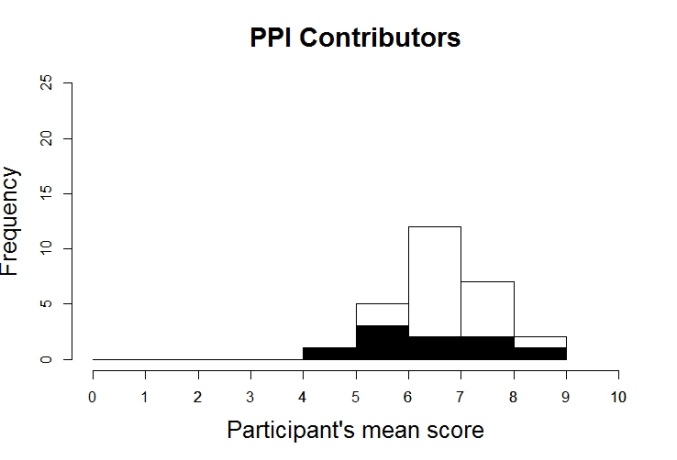 | 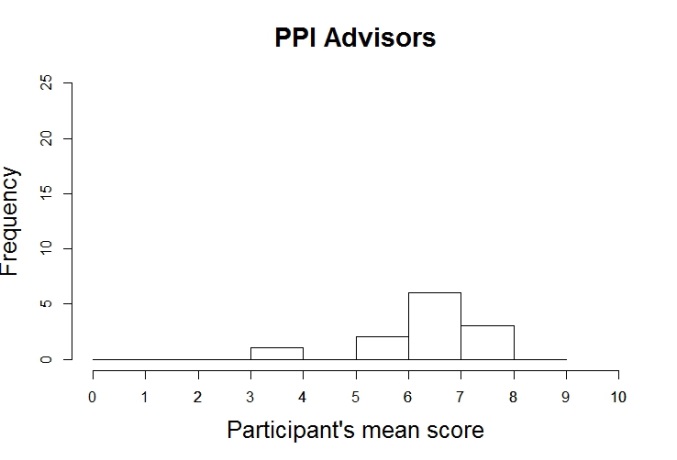 |
| --- | --- | --- |
| 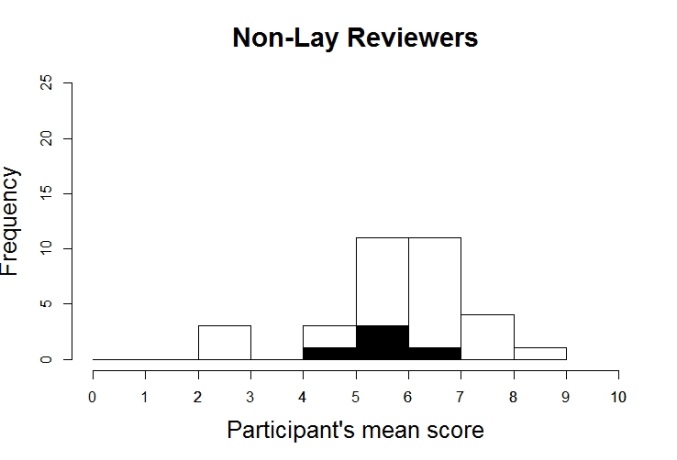 | 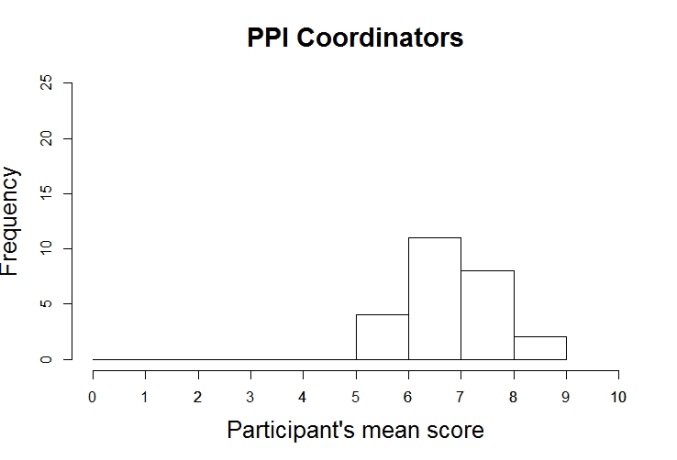 | 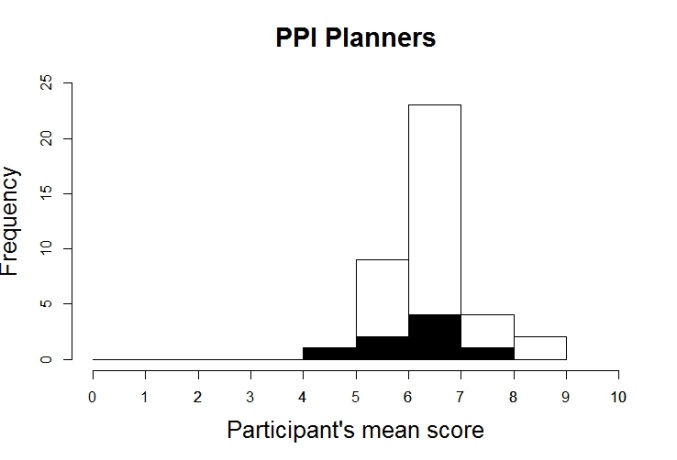 |
| 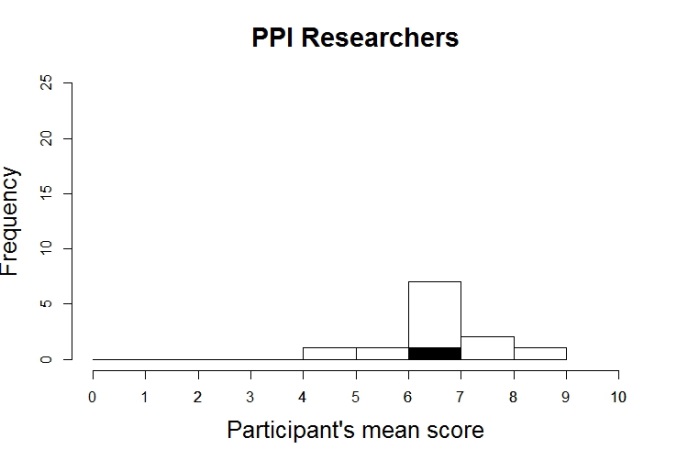 |  |  |
